# Retraction: *Fasciola hepatica* excretory-secretory products attenuate demyelination and reduce neuroinflammation in the Cuprizone –induced multiple sclerosis model

**DOI:** 10.1371/journal.pone.0362444

**Published:** 2026-11-10

**Authors:** The PLOS One Editors

Following the publication of this article [1], the corresponding author requested a correction to Fig 6C and 6D, after concerns were raised about unusually repetitive features appearing in these panels. The corresponding author stated that internal microscope-generated labels were removed from the Fig 6C and 6D image by covering the labelled areas with a portion of the adjacent image. They provided what they described as an original unedited image underlying Fig 6D, and a re-stained and reimaged panel for Fig 6C.

During editorial assessment, additional repetitive features were observed in Figs 6A and 6B. In response to these observations, the corresponding author provided what they described as original histopathology images underlying the published panels. However, the original images provided were not an exact match with the published figure, and repetitive features were also observed in areas of the published panel that did not have any labels in the original data, calling into question the integrity and reliability of the published results.

In light of these concerns, the *PLOS One* Editors retract this article.

AM and MSabzalizadeh did not agree with the retraction. KAK and MShabani responded but expressed neither agreement nor disagreement with the editorial decision. SMM, AK, and MFH either did not respond directly or could not be reached.
